# Synopsis of Crown and Bridge Work

**Published:** 1900-02

**Authors:** F. S. Casper

**Affiliations:** Austin, Texas


### Dental Department.

F. S. CASPER, D. D. S., AUSTIN, TEXAS.

### Synopsis of Crown and Bridge Work.

There is no doubt that crown and bridge work is no longer an experiment. It has played a successful part in the mechanical branch of dentistry since its advent, and has progressed more rapidly than any other branch of the profession. The pernicious practice of professional quacks, charlatans and montebanks, of applying bridges to every conceivable place, and in all kinds of manner and conditions, has been productive of a great amount of adverse criticism. Notwithstanding the abuses it has encountered, it has been a great blessing to many, and will continue to be in the hands of intelligent, competent dentists.

As in every other new departure it has its "Josiahs" and in the majority of cases of failure; the cause can be attributed to an improper consideration of the case in hand.

In all cases of bridge work the first requisite should be, to so construct the appliance that every part is easy of access to be cleansed of all foreign substances, which may have found lodgment from the food. If this cannot be done, the appliance will be an absolute failure.

Secondly, the material to be selected must be of such quality that will not tarnish or corrode, and it must be ductile with a certain amount of elasticity. Gold, platinum and iridium have been successfully employed, separately and in conjunction, but if gold alone is used it should be 22 karat fine, soldered with 20 karat solder.

Thirdly, the teeth upon which the bridge is to rest must be firm in the maxillary bone, and free from the ravages of pyorrhea alveolaris, and of such an inclination as to allow of the bridge being set when completed. The articulation must be carefully considered, a disregard of this particular will often insure failure. Whenever it is practicable to do so, a removable bridge should be constructed, as it has the preference over the permanent, in that it can be removed and cleansed when required.

As no two cases are exactly similar, no definite rule can be laid down for the guidance of the student. "Necessity, the mother of invention," will come to his rescue in difficult cases.

---

In the proceedings of the Minnesota Dental Association, discussing the best treatment for root filling, Dr. C. A. Van Duzee said: "I agree with Dr. Walls in the main; I want to emphasize and modify in the particulars. I want to warn any younger members of the society against placing too much faith in mumifying the living tissue in the mouth for any purpose. The use of alcohol in all this work, I think, should be emphasized. I do not think we have an agent as valuable as alcohol. I prefer oil of cassia as an oil dressing. I do not think it advisable to use it in front teeth, because it will turn the teeth brown. I do not believe carbolic acid has any place in the office. Prefer camphophenique. The canal of the root should be flooded with same. Chloro-percha has been a failure in my hands, believe in oxide of zinc paste as a root filling."

The use of chloro-percha has never proved attractive to us, as we believe that the hydrogen contained in the fluids of the mouth, combining with the sulphur in the chloro-percha, might produce an unsavory odor. Only an idea you know!

---

A Substitute for the Diamond Disk.—A small disk of thin copper, used with water or oil, will cut as perfectly as a diamond disk, and even more quickly.—*Dr. Roberts in International Dental Journal.*

---

Apples.—The apple is such a common fruit that few persons are familiar with its remarkably efficacious medicinal properties. Everybody ought to know that the very best thing he can do is to eat apples just before going to bed. The apple is excellent brain food, because it has more phosphoric acid, in an easily digested shape, than any other fruit known. It excites the action of the liver, promotes sound and healthy sleep, and thoroughly disinfects the mouth. It also agglutinates the surplus acids of the stomach, helps the kidney secretion, and prevents calculus growth, while it obviates indigestion, and is one of the best preventives of diseases of the throat. Next to lemon and orange, it is also the best antidote for the curing of persons addicted to the alcohol and opium habit.—*People's Health Journal, Chicago.*

---

Treating Sensitive Dentine.—In sensitive dentine, when the patients are extremely timid, dissolve a crystal of cocaine with a drop of carbolic acid, dry the cavity and apply the cocaine pigment on a small pledget of cotton. Protect the application from moisture; leave it for fifteen to twenty minutes when you can proceed, using sharp excavators first. It may be necessary to make the second application.

---

Annealing Gold.—You will never appreciate the true working qualities of cohesive gold until you quit passing it in the flame of the lamp. Use a sheet of mica or an annealing tray. Don't be penurious. The good effects will pay for the difference.—*Dr. W. H. Weaver, Dental Weekly.*

---

Subscribe for the Red-Back.—We mailed a large number of sample copies to dentists in different parts of the State, and would like to continue to send copies of the Journal to them, but the printer wishes to know who will pay the freight? This is a significant question.

